# A Kalman Filter for Nonlinear Attitude Estimation Using Time Variable Matrices and Quaternions

**DOI:** 10.3390/s20236731

**Published:** 2020-11-25

**Authors:** Álvaro Deibe, José Augusto Antón Nacimiento, Jesús Cardenal, Fernando López Peña

**Affiliations:** 1 Integrated Group for Engineering Research, University of A Coruña, Mendizábal s/n, 15403 Ferrol, Spain; flop@udc.es; 2 Observatory for Design and Innovation in Mobility, Transportation and Automotion, University of A Coruña, Mendizábal s/n, 15403 Ferrol, Spain; jaan@udc.es (J.A.A.N.); jcarde@udc.es (J.C.); 3 Center for Information and Communications Technology Research (CITIC), University of A Coruña, 15001 A Coruña, Spain

**Keywords:** Kalman Filter, attitude estimation, IMU, AHRS, quaternions

## Abstract

The nonlinear problem of sensing the attitude of a solid body is solved by a novel implementation of the Kalman Filter. This implementation combines the use of quaternions to represent attitudes, time-varying matrices to model the dynamic behavior of the process and a particular state vector. This vector was explicitly created from measurable physical quantities, which can be estimated from the filter input and output. The specifically designed arrangement of these three elements and the way they are combined allow the proposed attitude estimator to be formulated following a classical Kalman Filter approach. The result is a novel estimator that preserves the simplicity of the original Kalman formulation and avoids the explicit calculation of Jacobian matrices in each iteration or the evaluation of augmented state vectors.

## 1. Introduction

Inertial sensors (in particular, accelerometers and gyroscopes) are among the most common types of devices used for estimating the orientation of a solid body. Ideally, the orientation of a body can be calculated by integrating over time the signal from a three-axis gyroscope. However, the inherent bias and drift of the sensor introduce errors that increase throughout the integration process. This problem is more serious for low-cost Microelectromechanical System (MEMS) gyroscopes, which are more prone to drift and bias than larger and more expensive gyroscopes [1].

**Figure 1:**
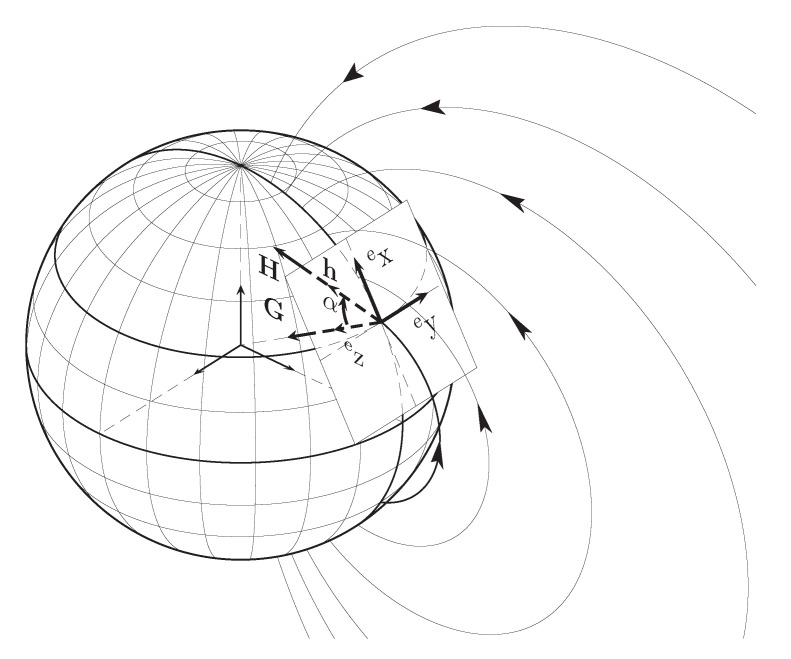
Earth reference system.

Magnetic sensors are also commonly used to measure the Earth’s local magnetic field. Combining inertial and magnetic sensors provides redundant information about the attitude of the body, including, but not limited to, information about two vectors that correspond to the Earth’s magnetic and gravitational fields (H and G in Figure 1). Several methods have been proposed to solve the estimation of the orientation of a body from two known vectors (for reviews, see [2,3]).

The Earth’s magnetic field vector, although variable with time, altitude, latitude and longitude, can be considered invariable in nearby surroundings. Thus, the Earth’s magnetic field measurement can be used to reduce the drift that many Inertial Measurement Unit (IMU) present around the vertical axis. The device resulting from the addition of a triaxial magnetometer to an IMU is usually known as a Magnetic, Angular Rate and Gravity (MARG) sensor. Under certain circumstances, it is possible to make a non-drifting attitude estimator based on a MARG sensor. A unit combining a MARG sensor with the electronics running the estimation algorithm is commonly called Attitude and Heading Reference System (AHRS). In modern outdoor applications, they are often combined with GNSS measurements, as in [4].

Most AHRSs have severe constraints concerning their size, weight and energy consumption and have limited computational power [5]. The estimators implemented in these systems need to be designed to comply with these restrictions. This is the case of the present approach, which achieves simplicity and low computational cost through the use of quaternions, variable time matrices and a particular state vector construct. The main goal is that, when estimating the attitude, nonlinear transformations due to rotations and changes of reference system can be written as matrix–vector products compatible with the original KF formulation. Some approaches for attitude estimation based on Linear Kalman Filter have been adopted by other authors, e.g. Ligorio and Sabatini [6].

As with quaternions, the use in KF of specific state vectors and time-varying matrices is not new. For example, Zhu and Zhou [2] used a state vector produced by accelerometer and magnetometer measurements. Gebre-Egziabher et al. [3] made use of a time-varying Kalman Filter implementation for gyro-free quaternion-based attitude determination. The main contribution of the current proposal is the use and combination of the chosen state-vector, quaternions and time-varying matrices.

## 2. Background

### 2.1. Orientation and Quaternions

Quaternions can be used to perform changes in attitude or system of reference through simple scalar addition and multiplication operations. This property allows writing these changes as matrix–vector products, which turns out to be essential for the particular formulation of the present method.

In addition, quaternions display some favorable characteristics: they do not present gimbal-locks and they produce a uniform approximation of attitude representation. Moreover, quaternion-based algorithms do not need to compute trigonometric functions. This characteristic highly reduces their computational cost, and it has been widely used in orientation algorithms, not only those based on KF as in [7] but also in others, such as complementary filters [8]. That is also taken advantage of in the present approach, although it is not an essential factor in its operation.

Appendix A contains a brief explanation of basic quaternion notation as it is used throughout this work.

### 2.2. Kalman Attitude Estimation

Kalman Filters (KF) are one of the most commonly used tools to solve the attitude estimation problem. This is probably because the KF approach can make use of every piece of information available to solve the problem, i.e., the dynamic model of the system, sensors data and the probabilistic footprint both of sensor signals and algorithm numerical behavior. Besides, the original KF turns out to be optimal and numerically efficient [9].

A discrete-time process can be described by the equations:

(1) $$\begin{array}{cc} x_{k} & =\Phi_{k}x_{k-1}+Bu_{k-1}+w_{k-1} \end{array}$$

(2) $$\begin{array}{cc} z_{k} & =H_{k}x_{k}+v_{k} \end{array}$$

Equation (1) is a stochastic difference equation that models the discrete-time process and serves to estimate the state vector x. Equation (2) describes the relationship between x and the measurement vector z. Vectors w and v are random variables that represent, respectively, process and measurement noise. They are assumed to be white, uncorrelated and having normal probability distributions:

(3) $$p\left(w\right)=N\left(0,Q\right),\;p\left(v\right)=N\left(0,R\right)$$

Matrix $B_{k}$ in Equation (1) is used to relate external actions $u_{k-1}$ to the state vector. However, in the present work, $u_{k}$ is always considered void, thus $B_{k}u_{k-1}$ is not present.

The original KF formulation [9] begins with a prediction or time update stage, which can be formulated as:

(4) $$\begin{array}{cc} {\hat{x}}_{k}^{-} & =\Phi_{k}{\hat{x}}_{k-1}\\ P_{k}^{-} & =\Phi_{k}P_{k-1}\Phi_{k}^{T}+Q_{k} \end{array}$$

followed by a correction or measurement update stage:

(5) $$\begin{array}{cc} K_{k} & =P_{k}^{-}H_{k}^{T}{\left(H_{k}P_{k}^{-}H_{k}^{T}+R_{k}\right)}^{-1}\\ {\hat{x}}_{k} & ={\hat{x}}_{k}^{-}+K_{k}\left(z_{k}-H_{k}{\hat{x}}_{k}^{-}\right)\\ P_{k} & =\left(I-K_{k}H_{k}\right)P_{k}^{-} \end{array}$$

Since the system model is quite often nonlinear, different modifications to the KF algorithm have been proposed to handle nonlinearities. These modifications usually cause KF to lose its optimality and, to some extent, its simplicity and efficiency. The Extended Kalman Filter (EKF) and Sigma Point Kalman Filter (SPKF) family are among the most common of these KF modifications.

The original KF algorithm [9] recursively estimates process state vector $x_{k}$ at any iteration *k* from its previous value, $x_{k-1}$, following a prediction–correction scheme. On the other hand, the EKF algorithm uses nonlinear functions $f(x_{k-i},u_{k})$ and $h(x_{k})$ instead of matrices.

Although being very similar to KF, some facts make EKF different. First, as f and h are not linear, EKF no longer meets the KF optimality conditions. Second, the use of these nonlinear functions may compromise the algorithm convergence. Third, it is assumed that $x_{k}$ is a Gaussian random variable (GRV), but, when evaluated by nonlinear functions f and h, it loses its GRV condition. Furthermore, at each step of the iteration, both Jacobian matrices used in the process and the nonlinear functions f and h must be evaluated. All these make the EKF computationally more expensive than the KF.

One possible way to overcome the problem of not preserving the GRV condition of x is by making use of a sample of characteristic points in the state space. These points are known as Sigma Points. Different methods have been proposed to sample representative sigma point sets, which result in new non-derivative Kalman Filters: the Unscented Kalman Filters (UKF) [10], the Central Difference Kalman Filters (CDF) [11] or the Divided Difference Filter (DDF) [12], among others. All these filters have been grouped into the Sigma-Point Kalman Filter (SPKF) family [13].

One of the main ideas behind SPKF is to increase the state vector x, of size *L*, by including process noises, making it grow to a size of 2L+1. This augmented vector is sampled 2L+1 times, to produce the sigma points. Then, these sigma points are propagated through the nonlinear equations of the process. This methodology eliminates the propagation of GRVs through the nonlinear functions present in the process, avoiding the costly computation of EKF Jacobian Matrices. However, sampling sigma points and propagating them through these nonlinear functions is also computationally expensive. This results in a suboptimal algorithm that is more complex than the original KF.

The proposed algorithm avoids these drawbacks by dealing with these nonlinearities in a simple and innovative way, as shown in the following section.

The purpose of what is presented in the rest of this document is to describe the bare filter and show its intrinsic behavior. Therefore, while possible, no acceleration restrictions are imposed, and no data from other sensors are provided. Under these conditions, the acceleration or attitude is expected to drift over time. This drift can be seen in Section 4, where the experimental results are discussed.

## 3. Proposed Filter Algorithm

A preliminary version of the attitude estimation algorithm described in this section is sketched in [14]. The algorithm follows the original KF approach [9], thus it is formulated as in Equations (4) and (5).

In the present case, the two stages represented by these equations are preceded by an initial stage to evaluate the matrices $\Phi_{k}$ and $H_{k}$ and followed by a final normalization stage, which guarantees that the orientation quaternion remains unit, as will be explained later.

Two reference systems are used: the body reference system, attached to the tracked body, and the Earth reference system. The latter has its *z* axis ($e_{z}$) pointing towards the Earth center, the *x* axis ($e_{x}$) oriented towards the magnetic north and the *y* axis ($e_{y}$) towards the magnetic east, as shown in Figure 1.

The main components of the estimator are described in the following.

### 3.1. State Vector

The state vector $x_{k}$ has been chosen to allow writing prediction and correction stages as matrix–vector products. This allows maintaining the original KF formulation. It is composed of measurable physical magnitudes: accelerations, orientation and angular velocities. For time step *k*, it can be written as:

(6) $${}^{e}x_{k}={\left(\begin{array}{ccc} {}^{e}a_{k} & {}^{e}q_{k} & {}^{b}v_{r,k} \end{array}\right)}^{T}$$

where

- ${}^{e}a_{k}={\left(\begin{array}{ccc} a_{x} & a_{y} & a_{z} \end{array}\right)}^{T}$ is the acceleration vector expressed in Earth coordinates. It takes into account external accelerations due to changes in velocity. This approach differs from most MARG attitude estimators based on Kalman Filter [15], where the average value of external accelerations is usually set to zero.
- ${}^{e}q_{k}={\left(\begin{array}{cccc} q_{1} & q_{2} & q_{3} & q_{4} \end{array}\right)}^{T}$ is a unit quaternion representing body orientation expressed in earth coordinates. It is used here to change between body and Earth reference systems (see Appendix A). The last stage of the filter algorithm includes the necessary corrections to enforce that this quaternion remains unitary.
- ${}^{b}v_{r,k}={\left(\begin{array}{ccc} v_{x} & v_{y} & v_{z} \end{array}\right)}^{T}$ is the vectorial part of the rotation quaternion ${}^{b}q_{r}=\left(\omega_{0},{}^{b}v_{r}\right)$ at time step *k*. This quaternion represents the orientation change in body coordinates. It should be noted that $\omega_{0}$, the scalar part of ${}^{b}q_{r}$ is not needed, provided that this quaternion remains unitary.

As shown below, matrices $\Phi_{k}$ and $H_{k}$ in Equations (4) and (5) include vectors that need to be reoriented or expressed in a different base. However, the state vector includes information on orientation and rotation in quaternion ${}^{e}q_{k}$ and vector ${}^{b}v_{r,k}$, respectively. Therefore, these changes in orientation, or base changes, occur directly when the state vector is multiplied by any of these matrices.

### 3.2. Measurement Vector

Vector $z_{k}$ is the measurement vector. It contains information obtained from the sensors at time *k*, expressed in the local (body) reference system:

(7) $$z_{k}={\left(\begin{array}{ccc} {}^{b}a_{k} & {}^{b}m_{k} & {}^{b}\omega_{k} \end{array}\right)}^{T}$$

where ${}^{b}a_{k}$ are the measurements of the linear accelerations, obtained from a triaxial accelerometer; ${}^{b}m_{k}$ contains the measurements of the Earth’s magnetic field along the three orthogonal axes of the body reference system; and ${}^{b}\omega_{k}$ are the three angular velocity measurements obtained from a triaxial rate-gyro.

In the remainder of this section, the subscript *k* is omitted for convenience, as long as this cannot lead to misinterpretation.

### 3.3. The Process Model

Matrix Φ in Equation (4) is built as block diagonal, and it is used to perform the prediction stage:

(8) $$\begin{array}{c} \Phi =\mathrm{diag}(\Phi_{11}\;\Phi_{22}\;\Phi_{33}) \end{array}$$

Each block in Φ has a counterpart in the state vector:

- Acceleration: In the prediction phase, it is assumed that acceleration remains constant (there is no direct measure of external forces), which leads to:
  (9) $$\Phi_{11}=I_{3\times 3}\;\Rightarrow \;{}^{e}a_{k+1}\leftarrow {}^{e}a_{k}$$
- Orientation quaternion: In the prediction phase, it is also assumed that the angular velocity remains constant, so the orientation using relation Equation (A10) in Appendix A changes accordingly:
  (10) $$\Phi_{22}\;\cdot \;{}^{e}q\;={}^{e}q\;⊗\left(\begin{array}{c} \omega_{0}\\ {}^{b}v_{r} \end{array}\right)=\left(\omega_{0}\;\cdot \;I_{44}+\Omega \right)\;\cdot \;{}^{e}q\;$$
  Thus, the block $\Phi_{22}$ of the transition matrix takes the following form:
  (11) $$\Phi_{22}=\omega_{0}I_{44}+\Omega \;\Rightarrow \;{}^{e}q_{k+1}\;\leftarrow \Phi_{22}\;\cdot \;{}^{e}q_{k}$$
  where matrix Ω depends on the rotation vector ${}^{b}v_{r}$ (Equation (A12) in Appendix A).
- Rotation quaternion: As the angular velocity is assumed to be constant, the rotation quaternion remains invariant. Thus, $\Phi_{33}$ becomes:
  (12) $$\Phi_{33}=I_{3\times 3}\;\Rightarrow \;{}^{b}v_{r,k+1}\leftarrow {}^{b}v_{r,k}$$

It is important to note that, given the way that the transition matrix Φ has been built, it fits into the structure of a linear KF. The fact that Φ is a time variable matrix, combined with the use of quaternions and the described state vector, is what allows handling a nonlinear problem using a linear scheme.

### 3.4. Covariance Matrices

Because Φ is block diagonal, the process noise covariance matrix is also block diagonal:

(13) $$Q=\mathrm{diag}(Q_{a}\;Q_{q}\;Q_{r})$$

The blocks in Q correspond to acceleration ${}^{e}a$, orientation quaternion ${}^{e}q_{k}$ and rotation quaternion ${}^{b}v_{r}$ (see Appendix B).

### 3.5. The Observation Model

Matrix H is used to relate the sensors measurement vector to the state vector through product z=H · x:

(14) $$\left(\begin{array}{c} {}^{b}a\\ {}^{b}m\\ {}^{b}\omega \end{array}\right)=\left(\begin{array}{ccc} H_{11} & H_{12} & 0\\ 0 & H_{22} & 0\\ 0 & 0 & H_{33} \end{array}\right)\;\cdot \;\left(\begin{array}{c} {}^{e}a\\ {}^{e}q\\ {}^{b}v_{r} \end{array}\right)$$

Matrices $H_{11}$ and $H_{12}$ depend on the orientation ${}^{b}q$ (see Appendix C). The gravity vector $g\;\cdot \;e_{z}$ and the acceleration vector ${}^{e}a$ are expressed in the inertial reference frame. By performing a change of basis on Equation (A4), the accelerometer measurement vector can be constructed as:

(15) $$\left(\begin{array}{c} 0\\ {}^{b}a \end{array}\right)={}^{e}q*\;⊗\left(\begin{array}{c} 0\\ {}^{e}a \end{array}\right)⊗{}^{e}q\;-g\;\cdot \;{}^{e}q*\;⊗\left(\begin{array}{c} 0\\ e_{z} \end{array}\right)⊗{}^{e}q$$

Gravity, *g*, is measured at the beginning of each test and is assumed to be constant throughout the experiment. Using Equation (A10) in Appendix A, Equation (15) can be expressed as the product of a (3×3) matrix by a vector, in the first case, and as a (3×4) matrix by a quaternion, in the second:

(16) $${}^{b}a=H_{11}\;\cdot \;{}^{e}a+H_{12}\;\cdot \;{}^{e}q\;$$

Matrix $H_{22}$ (see Appendix C) is used to perform the estimation of the magnetometer output. This estimation can be expressed as:

(17) $${}^{b}m=h\;\cdot \;{}^{e}q*\;⊗\left(\sin\alpha \;\left(\begin{array}{c} 0\\ e_{x} \end{array}\right)+\cos\alpha \;\left(\begin{array}{c} 0\\ e_{z} \end{array}\right)\right)⊗{}^{e}q$$

where α is the angle between the local magnetic and gravity fields and *h* is the magnetic field modulus. Both are measured at the beginning of each experiment and assumed to remain constant. This equation can be expressed as a matrix by quaternion product:

(18) $${}^{b}m=H_{22}\;\cdot \;{}^{e}q$$

Matrix $H_{33}$ performs a prediction on the rate-gyro readings as a function of the rotation velocity according to ${}^{b}\omega =H_{33}{}^{b}v_{r}$. In this case, both vectors are expressed in the local reference frame, so quaternion ${}^{e}q$ is not needed. Using Equation (A8) in Appendix A, matrix $H_{33}$ can be written as:

(19) $$H_{33}=\frac{2}{\Delta t}I_{3\times 3}$$

The primary purpose of this work is to show the viability of the bare estimator. That is why typical errors of the sensors such as drift of the rate-gyros have been considered negligible. However, the estimator can be modified to deal with these issues. Matrix $H_{33}$ can be reformulated such that it can model the drift of rate-gyros. In addition, after incorporating the temperature measurement to the state vector, matrix H could be modified to deal with the temperature drift of all of the sensors.

As it happens with Φ, block matrices $H_{ij}$ are time variable.

## 4. Experimental Results

As discussed in previous sections, the proposed new filter formulation is simpler and computationally less demanding than other common KF implementations for nonlinear estimation. This feature will be of interest in many implementations, particularly those related to AHRS. The next step is to verify the operation of this filter in some test applications. Therefore, in this section, the results of numerical and real test cases are discussed. The objective is to empirically compare the proposed filter with other KFs, in terms of effectiveness, precision and accuracy.

The numerical test case presents a simulated double pendulum maneuver in which there are very sudden changes in body orientation. The result is obtained numerically, and the consequent accelerations, angular velocities and magnetic vectors are calculated and taken as ground truth, both directly and after adding an amount of 3D white noise similar to the one characterizing MARG sensors. Even though the actual movement is two dimensional, no constraints are imposed on the virtual sensors. They perform their measurements as if the bodies were free to move in full 3D. In this situation, some accumulative drift is expected, driving the estimated maneuver out of its plane.

The second test case consists of a real maneuver tracking of a solid, instrumented with real MARG sensors. The proposed attitude estimator is fed with measurement data taken from these sensors. The estimated orientation obtained with this filter is qualitatively compared to the real one and also to the estimations obtained with other published filters.

### 4.1. Double Pendulum

**Figure 2:**
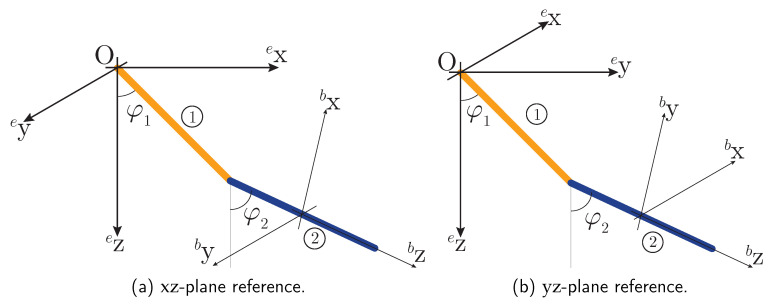
Reference systems for the double pendulum case study.

A double compound pendulum (Figure 2) is considered. Both links are revolute joints as a planar motion is modeled. Its inertial reference system is located on the Earth’s surface, having x, y and z axes pointing north, east and down, respectively. The pendulum is assumed to be ideal, without friction or any other cause that could dissipate energy and evolves freely in a vertical plane under the action of gravity. The local reference system is chosen so that it is aligned with the inertial one when the mechanism is at rest in stable dynamic equilibrium, i.e., having both bars aligned and pointing down.

Considering $\varphi_{1}$ and $\varphi_{2}$ as generalized coordinates, the Lagrange equations lead to the following second-order system of ordinary differential equations (ODE).

(20) $$\begin{array}{c} \left[\begin{array}{cc} m_{1}L_{1}^{2}+I_{1}+4m_{2}L_{1}^{2} & 2m_{2}L_{1}L_{2}\cos\left(\varphi_{1}-\varphi_{2}\right)\\ 2m_{2}L_{1}L_{2}\cos\left(\varphi_{1}-\varphi_{2}\right) & m_{2}L_{2}^{2}+I_{2} \end{array}\right]\left\{\begin{array}{c} {\ddot{\varphi }}_{1}\\ {\ddot{\varphi }}_{2} \end{array}\right\}=\\ \left\{\begin{array}{c} -2m_{2}L_{1}L_{2}{\dot{\varphi }}_{2}^{2}\sin\left(\varphi_{1}-\varphi_{2}\right)-\left(m_{1}+2m_{2}\right)gL_{1}\sin\varphi_{1}\\ 2m_{2}L_{1}L_{2}{\dot{\varphi }}_{1}^{2}\sin\left(\varphi_{1}-\varphi_{2}\right)-m_{2}gL_{2}\sin\varphi_{2} \end{array}\right\} \end{array}$$

where *g* is the acceleration of gravity; $m_{1}$, $I_{1}$ and $L_{1}$ are, respectively, the mass, inertia moment and semi-length of bar 1; and $m_{2}$, $I_{2}$ and $L_{2}$ are the mass, inertia moment and semi-length of bar 2.

This ODE system is solved numerically with enough precision to be considered as ground truth. This makes it also possible to generate a set of synthetic measurements supposedly taken by a set of virtual MARG sensors located at the mass center of the bar labeled 2 in Figure 2. These virtual measurements of acceleration, angular velocity and magnetic field are inputs to the filter. The resulting orientation estimation is compared then to the ground truth to provide evidence on the proposed filter fitness.

The masses and semi-lengths of pendulum elements 1 and 2 are set to be equal to unity ($L_{1}=L_{2}=1\;m$, $m_{1}=m_{2}=1\;kg$). The multibody system evolves from an initial still position, with both bars placed horizontally. The simulation provides a set of values of $\varphi_{1}$, $\varphi_{2}$, ${\dot{\varphi }}_{1}$ and ${\dot{\varphi }}_{2}$ for the first 10 s of the simulated pendulum motion. From these values, the measured values acquired by the virtual MARG sensors are calculated without error (except computational round-off).

This section presents the results of two instances of the experiment. In the first one, clean virtual measurements are used directly, while, in the second one, Gaussian white noise is added to these measurements. This added noise has zero mean and a variance characteristic of each type of triaxial sensor making up the MARG: accelerometer, rate-gyro and magnetometer (see Table 1). These values have been previously obtained experimentally [14] for a particular MARG sensor.

Both instances of the experiment have been performed in two different ways. The first run takes place in the xz-plane. This plane contains the gravity and magnetic field vectors, thus virtual sensors attached to bar 2 do not have any input out of this plane (see Figure 2). In the second run, the planar movement of double pendulum takes place in the yz-plane, which does not contain the magnetic field vector, so that virtual magnetometers perceive inputs with non-zero values in the three axes.

Although the proposed filter uses quaternion algebra, for clarity, Euler’s parameters ϕ, θ and ψ are used to present the results. These parameters correspond to rotations around the ${}^{e}x$, ${}^{e}y$ and ${}^{e}z$ axes. The angle $\varphi_{2}$ that defines the orientation of the second bar coincides with Euler’s θ parameter in the first case, while it coincides with the ϕ parameter in the second. In the test variants where sensor noise is added, since this noise is 3D, all sensors produce non-zero outputs on their three axes.

**Figure 3:**
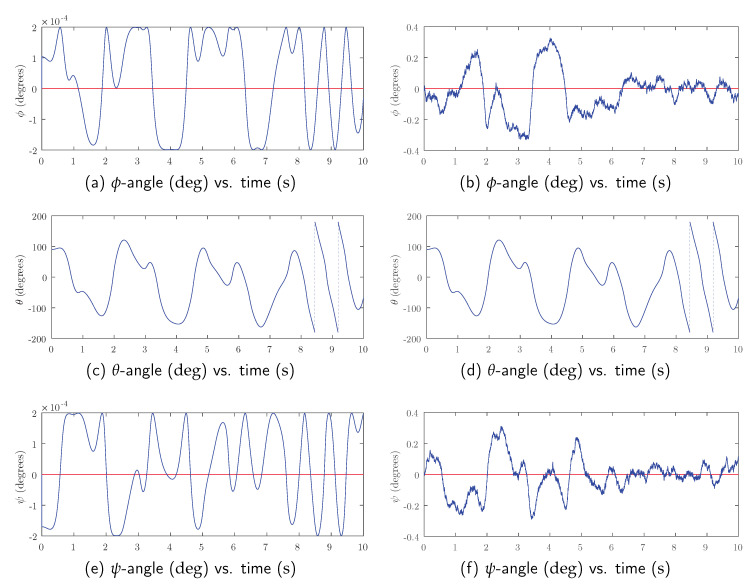
Simulation of the dynamics of the double pendulum on the xz-plane: Euler parameters calculated by numerical integration (red line) and estimated using the proposed filter when virtual sensor data have no noise (left) and when they have noise (right).

Figure 3 summarizes the results when the simulation is carried out in the xz-plane. It includes a comparison between the Euler’s parameters obtained as ground truth and those provided by the proposed estimator, without (left column) and with added noise (right column). Figure 3c,d represents values of angle $\varphi_{2}$ corresponding to the true motion. The differences between ground truth (red line) and estimated values (blue line) of the angle $\varphi_{2}$ are so small that both lines are superimposed in these two figures. The rest of the subfigures present the out-of-plane perceived movement. As this should be null, the ground truth remains equal to zero. However, as expected, the estimator produces non-zero outputs here. Nevertheless, these outputs remain within very contained values, in the order of $1\times 10^{-4}$ deg without noise and $1\times 10^{-1}$ deg with added noise.

Table 2 summarizes the differences between angular estimation and ground truth in all test cases. As can be seen, the mean value of these differences is always close to zero. Besides, the variance of errors in the estimation of angle $\varphi_{2}$ rotated by bar 2 is in the order of $1\times 10^{-1}$ deg.

Gaussian noise appears to have a noticeable effect regarding the fake off-plane displacements produced by the estimator:

- In the noiseless case, there are differences between estimations in the xz-plane and the yz-plane. While the mean error can be considered negligible in both cases, it is closer to zero by two orders of magnitude when there are no out-of-plane measurements (xz-plane case).
- In the with noise case, the average of deviations between the expected and estimated values is approximately zero, while the standard deviation is of the same order of magnitude in both simulations (about a tenth of a degree).

Henceforth, it can be gathered that estimation errors that lead to out-of–plane movements are small oscillations that do not represent a continuous drift in any direction. For this reason, it can be concluded that the proposed orientation estimator behaves precisely and stably in this synthetic test.

### 4.2. Consecutive Decoupled Turn on Each Axis

The second test presents a true maneuver. The purpose of this example is to exhibit the ability of the proposed filter to track a real body movement and check its ability to follow a known maneuver quickly and stably.

In this experiment, a solid box is subjected to some independent and sequential turning maneuvers. It is instrumented with a MARG sensor that allows measuring acceleration, angular velocities and magnetic field over time. For this purpose, an AHRS device manufactured by Redshift Labs Pty Ltd. (formerly CH Robotics LLC) was selected [16]. Its core is an InvenSense MPU-9150, which includes a set of triaxial sensors (rate-gyro, accelerometer and compass).

**Figure 4:**
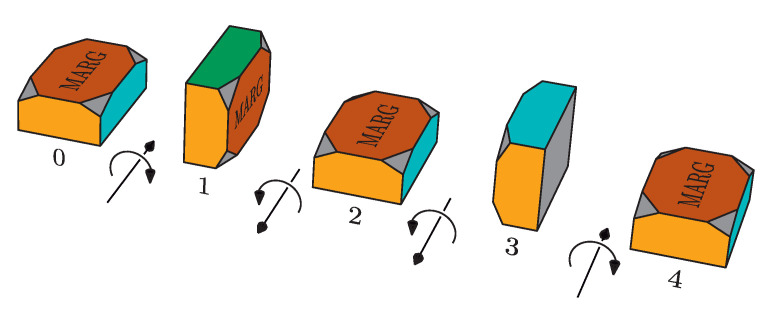
Schematic representation of turn maneuver in one axis.

The instrumented body is initially oriented so that the local and global reference frames coincide. As shown in Figure 4, each turning maneuver consists of a turn of π/2 rad, followed by a turn of −π rad and a final turn of π/2 rad that returns the body to its initial orientation. The complete experiment includes rotations around each of the axes of the frame of reference.

Each maneuver is separated from the next by a period of 5 s at rest and is performed at a speed of 1 s per turn. All movements are performed by hand and, consequently, the accuracy of both the rotated angle and timing is not guaranteed. However, during the idle periods, with values of ±π/2 rad, the box is lying on a flat horizontal surface and backed by a fixed vertical dihedral. Therefore, these idle intervals can be used as a reference. Similar orientation tests were performed by Farhangian and Landry [17] for probing an intermittent calibration technique of an EKF-based attitude determination algorithm.

**Figure 5:**
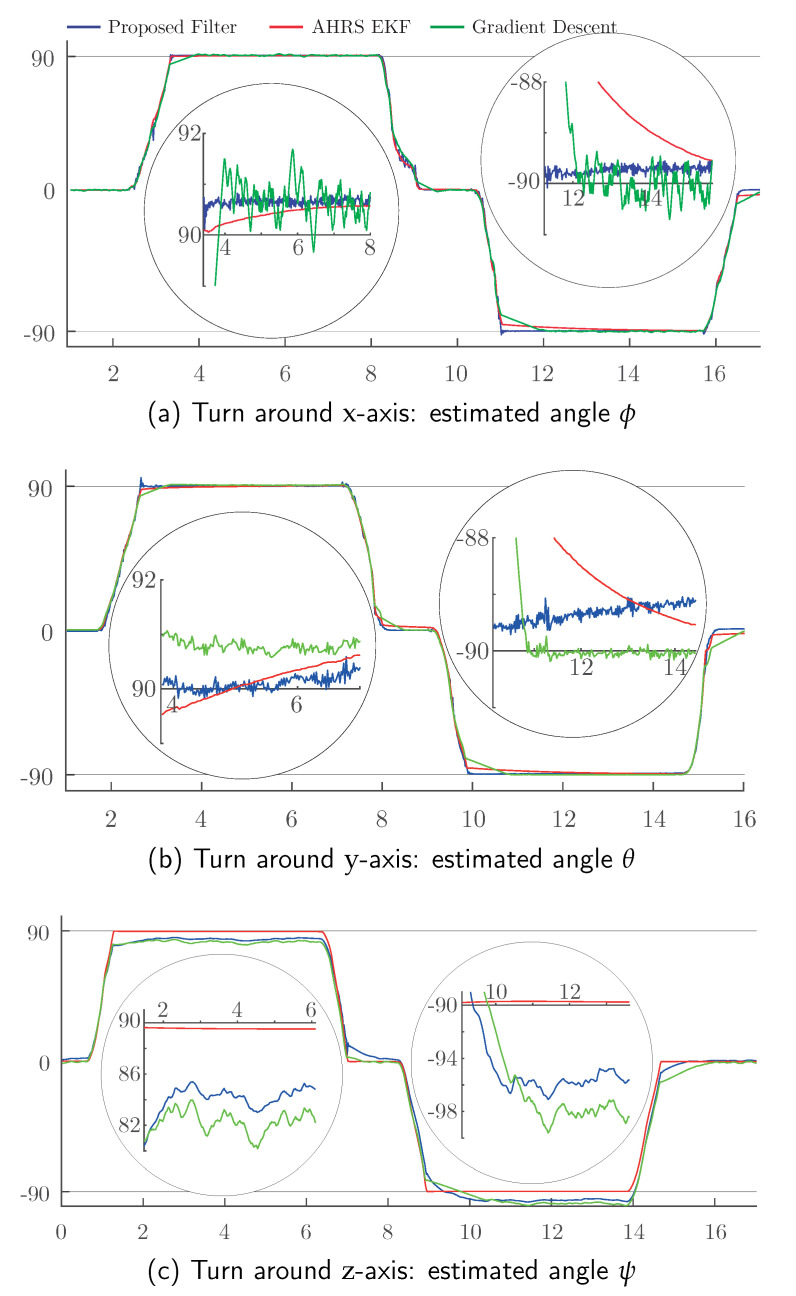
Turn around each axis: estimated angle (deg) vs. time (s).

The MARG raw datasets were collected during the maneuver and used to feed the proposed filter and two other orientation estimators: a proprietary EKF embedded in the AHRS microcontroller [16] and a filter based on a gradient descent algorithm proposed by Madgwick et al. [18,19]. A qualitative comparison between these three filters is shown in Figure 5.

Figure 5a–c presents the results of the estimated solid body orientation over time as computed by the embedded EKF, gradient descent and proposed filters. Each graph represents the estimated turned angle on each axis and includes two zoomed details at idle states in π/2 and −π/2.

In terms of promptness, stability and precision, it can be seen that the proposed estimator performs well. Figure 5, and especially Figure 5b, shows a rotational bias drift. This drift is the result of not having imposed any restrictions on acceleration. As indicated above, this type of correction has been avoided to allow a better view of the bare filter behavior.

A priori, the algorithm embedded into the AHRS should perform better than the gradient descent and the proposed estimator. First, the proprietary EKF operates at a frequency rate of 500 Hz (there is no information about its inner data acquisition frequency rate, which could be higher) while input data rate for both the proposed and the gradient descent estimators is of 50 Hz. Second, the embedded EKF has been factory tuned with an unpublished sensor characterization. Therefore, it has not been possible to include it in the other two algorithms. This lack of sensor characterization is more evident when performing a turn maneuver around the z axis because, in this case, the rate-gyro that measures angular velocity on this axis has less precision than the other two [16].

None of the three shown estimators is the best performer in every scenario, as can be seen from the six insets in Figure 5. However, it is noteworthy that, in some circumstances, such as in the second inset of Figure 5a, the proposed algorithm performs better than the proprietary, factory-tuned EKF. Besides, it is feasible to improve the behavior of the proposed estimator by making adjustments and fine-tuning it.

## 5. Conclusions

This work presents a new formulation and the corresponding algorithm for a Kalman Filter that is used to estimate the attitude of a rigid body when the input data come from MARG sensors.

The starting point for developing this algorithm is the original formulation of the Kalman Filter. The characterization of the rigid body orientation in space is achieved by a construction that uses quaternions instead of Euler angles. Two quaternions are used, one for orientation and another for rotational speed. A specific state vector made up of these two quaternions plus the acceleration vector allows expressing equations containing trigonometric functions as simple matrix by vector products. These matrices are time variable and allow modeling the nonlinear dynamic behavior of the process as a succession of linear steps within the KF.

For the execution of the resulting time variable Kalman Filter (TVKF), it is not necessary to calculate trigonometric functions, obtain Jacobians, or perform any type of complex mathematical operation. In addition since it is derived from the original KF formulation, the drawbacks of other nonlinear approaches such EKF or SPKF are avoided. Instead, in the current approach, the coefficients of the TVKF filter matrices are calculated at each time step by using simple algebraic operations. As a consequence, the filter seems to be well suited to be used in the code embedded in MARG sensors, given their limited computational resources.

This work intends to present this TVKF and show how it behaves. For the latter purpose, the results of two sets of tests are presented and discussed. For the first set, the simulated movement of a composite pendulum is used. This allows the numerical solution to the equations that govern this movement to be used as the fundamental truth. In the second set, a real case is considered. Here, the results obtained by the presented TVKF are compared to those obtained with two Kalman Filters: one is the commercial KF incorporated in the MARG sensor that was used and the other was obtained from the literature.

## Figures and Tables

**Table 1:** Sensor variance characterization.

Sensor [Units]	Without Noise	With Noise
Rate-gyro $\left[{\mathrm{rad}}^{2}/s^{2}\right]$	1 × 10${}^{-16}$	3.6 × 10${}^{-3}$
Accelerometer $\left[m^{2}/s^{4}\right]$	1 × 10${}^{-16}$	2 × 10${}^{-4}$
Magnetometer $\left[G^{2}\right]$	1 × 10${}^{-16}$	1 × 10${}^{-6}$

**Table 2:** Mean and RMS values of differences (deg) between expected and estimated angles.

Plane	Difference	Without Noise		With Noise	
	(deg)	Mean	RMS	Mean	RMS
	$\phi_{\epsilon }$	2.8 × 10${}^{-5}$	1.3 × 10${}^{-5}$	−9.6 × 10${}^{-3}$	0.12
xz	$\varphi_{2e}\;\equiv \;\theta_{\epsilon }$	−4.4 × 10${}^{-2}$	1.6 × 10${}^{-1}$	−4.4 × 10${}^{-2}$	0.16
	$\psi_{\epsilon }$	2.7 × 10${}^{-5}$	1.4 × 10${}^{-5}$	−2.5 × 10${}^{-2}$	0.14
	$\varphi_{2e}\;\equiv \;\phi_{\epsilon }$	2.0 × 10${}^{-2}$	7.1 × 10${}^{-2}$	−3.2 × 10${}^{-2}$	0.15
yz	$\theta_{\epsilon }$	8.1 × 10${}^{-4}$	1.0 × 10${}^{-1}$	−2.7 × 10${}^{-2}$	0.12
	$\psi_{\epsilon }$	5.0 × 10${}^{-3}$	1.0 × 10${}^{-1}$	−2.2 × 10${}^{-2}$	0.14

## Abbreviations

The following abbreviations are used in this manuscript:

AHRS	Attitude and Heading Reference System
CDF	Central Difference Filter
DDF	Divided Difference Filter
EKF	Extended Kalman Filter
GRV	Gaussian Random Variable
IMU	Inertial Measurement Unit
KF	Kalman Filter
MARG	Magnetic, Angular Rate, and Gravity
MEMS	Micro Electro Mechanical Sensor
ODE	Ordinary Differential Equation
SPKF	Sigma–Point Kalman Filter
TVKF	Time Variable Kalman Filter
UKF	Unscented Kalman Filter
VLKF	Variable Linear Kalman Filter

## Appendix A. Quaternion Notation

The following is a brief explanation of basic quaternion notation and expressions as they are used throughout this paper. In the quaternion

(A1) $$q\;={\left(q_{0}\;q_{1}\;q_{2}\;q_{3}\right)}^{T}={\left(w\;s^{T}\right)}^{T}$$

the first component, $q_{0}$, is the scalar part, while the next three, $q_{1},q_{2}$ and $q_{3}$, constitute the vector part, s.

Any unit quaternion can be used to represent a rotation, thus unit quaternion q can also be expressed as

(A2) $${}^{e}q\;={\left(\begin{array}{c} \cos\frac{\varphi }{2}\;\sin\frac{\varphi }{2}\;\cdot \;{}^{e}v^{T} \end{array}\right)}^{T}$$

where φ is the rotated angle and v is a unit vector defining the axis around which rotation takes place. The left superscript *e* denotes the Earth reference system, while the left superscript *b* denotes the body reference system. The rotated angle φ should fall within the interval [−π, π) so that the scalar part is always positive.

Quaternion ${}^{e}q$ can be used to rotate the axes of the inertial reference system (Earth) to align them to the body reference system. Thus, ${}^{e}q$ also represents the change of basis between these two reference systems. Thus, the components of any vector u in both reference systems are related as follows [20]:

(A3) $$\begin{array}{cc} \left(\begin{array}{c} 0\\ {}^{e}u \end{array}\right) & ={}^{e}q\;⊗\left(\begin{array}{c} 0\\ {}^{b}u \end{array}\right)⊗{}^{e}q* \end{array}$$

(A4) $$\begin{array}{cc} \left(\begin{array}{c} 0\\ {}^{b}u \end{array}\right) & ={}^{e}q*⊗\left(\begin{array}{c} 0\\ {}^{e}u \end{array}\right)⊗{}^{e}q \end{array}$$

where ⊗ and * denote quaternion product and conjugate, respectively. This relations can also be expressed as matrix–vector products [21]:

(A5) $$\begin{array}{c} {}^{e}u={}_{b}^{e}C\left({}^{e}q\;\right)\;\cdot \;{}^{b}u \end{array}$$

(A6) $$\begin{array}{c} {}^{b}u={}_{e}^{b}C\left({}^{e}q\;\right)\;\cdot \;{}^{e}u \end{array}$$

### Orientation Quaternion Derivative

A detailed description of the quaternion orientation derivative can be found in [22]. The derivative of orientation quaternion ${}^{e}q\;$ is given by the following expression:

(A7) $$\frac{d}{dt}{}^{e}q\;=\lim_{\Delta t\to 0}\frac{1}{\Delta t}\left({}^{e}q\;⊗{}^{b}q_{r}\;-{}^{e}q\right)$$

Assuming there was an orientation change in Δt, ${}^{b}q_{r}$ (evaluated in the body reference system, as indicated by the left “*b*” superscript) represents the rotation of orientation quaternion ${}^{e}q$ in this time step. As Δt→0, rotated angle δφ could be considered small enough to approximate ${}^{b}q_{r}$ as:

(A8) $${}^{b}q_{r}\;=\left(\begin{array}{c} \cos\frac{\delta \varphi }{2}\\ \sin\frac{\delta \varphi }{2}\;\cdot \;v \end{array}\right)≃\left(\begin{array}{c} 1\\ \frac{\Delta t}{2}\;{}^{b}\omega \end{array}\right)$$

where ${}^{b}\omega$ is the angular velocity measured in the body reference system. Introducing Equation (A8) into (A7) leads to:

(A9) $$\frac{d}{dt}\;{}^{e}q\;=\frac{1}{2}\;{}^{e}q\;⊗\left(\begin{array}{c} 0\\ {}^{b}\omega \end{array}\right)$$

Using the relationships

(A10) $$q\;⊗\left(\begin{array}{c} 0\\ \omega \end{array}\right)=\Omega \left(\omega \right)\;\cdot \;q\;=\Xi \left(q\right)\;\cdot \;\omega$$

Equation (A9) can be re-written in matrix form as follows:

(A11) $$\frac{d}{dt}\;{}^{e}q\;=\frac{1}{2}\;\Omega \left({}^{b}\omega \right)\;\cdot \;{}^{e}q$$

where matrices Ω(ω) and Ξ(q) are:

(A12) $$\Omega \left(\omega \right)=\left(\begin{array}{cccc} 0 & -\omega_{1} & -\omega_{2} & -\omega_{3}\\ \omega_{1} & 0 & \omega_{3} & -\omega_{2}\\ \omega_{2} & -\omega_{3} & 0 & \omega_{1}\\ \omega_{3} & \omega_{2} & -\omega_{1} & 0 \end{array}\right),\;\Xi \left(q\right)=\left(\begin{array}{ccc} -q_{1} & -q_{2} & -q_{3}\\ q_{0} & -q_{3} & q_{2}\\ q_{3} & q_{0} & -q_{1}\\ -q_{2} & q_{1} & q_{0} \end{array}\right)$$

The quaternion product is not commutative. However, product $q\;⊗{\left(0\;\omega \right)}^{T}$ in Equation (A9) can be written as a matrix×vector product using the Ω or Ξ matrices.

## Appendix B. Covariance Matrices

The parameterization of the covariance matrices, in the cases of acceleration ${}^{e}a$ and rotation quaternion ${}^{b}v_{r}$, uses two parameters whose values need to be adjusted:

(A13) $$\begin{array}{cc} Q_{a,k} & =\sigma_{a}^{2}\;\cdot \;I_{3\times 3} \end{array}$$

(A14) $$\begin{array}{cc} Q_{r,k} & =\sigma_{r}^{2}\;\cdot \;I_{3\times 3} \end{array}$$

In the case of orientation quaternion (${}^{e}q_{k}$), the covariance matrix can also be expressed in terms of rate-gyros variances. The measurements taken by the rate-gyros (obviating at this time the bias drift) can be modeled as:

(A15) $${}^{b}\omega =\overset{˘}{\omega }+v_{g}\;\Sigma_{g}=E\left(v_{g}v_{g}^{T}\right)=\sigma_{g}^{2}\;I_{3\times 3}$$

where $\overset{˘}{\omega }$ is the true angular velocity and ${}^{b}\omega$ is the angular velocity as measured by the rate-gyros. Vector $v_{g}$ is the part of the measurement noise given in Equation (A2) that is due to the rate-gyros, which has zero mean and a covariance matrix $\Sigma_{g}$.

Using this notation, replacing ${}^{b}\omega$ by $\overset{˘}{\omega }$ in Equation (A9) and using the relation given in Equation (A10), the process noise due to the orientation quaternion $w_{q}$ is given by:

(A16) $$w_{q,k}=-\frac{\Delta t}{2}\Xi \left({}^{e}q_{k}\right)\;\cdot \;v_{g,k}$$

Finally, the process noise covariance matrix $Q_{q,k}$ can be written as follows [23,24]:

(A17) $$Q_{q,k}={\frac{\Delta t}{4}}^{2}\;\Xi \left({}^{e}q_{k}\right)\;\Sigma_{g}\;\Xi {\left({}^{e}q_{k}\right)}^{T}={\frac{\Delta t}{4}}^{2}\;\sigma_{g}^{2}\;\left(tr\left(P_{q,k}\right)I_{4\times 4}-P_{q,k}\right)$$

where $P_{q,k}$ is the error covariance matrix of the quaternion component of the state vector.

## Appendix C. Matrix H Expression

Submatrices $H_{11}$, $H_{12}$ and $H_{22}$ in matrix H (Equation (14) in Section 3.5) can be written as follows:

(A18) $$H_{11}=\left(\begin{array}{ccc} 1-2\left(q_{2}^{2}+q_{3}^{2}\right) & 2\left(q_{0}q_{3}+q_{1}q_{2}\right) & 2\left(q_{1}q_{3}-q_{0}q_{2}\right)\\ 2\left(q_{1}q_{2}-q_{0}q_{3}\right) & 1-2\left(q_{1}^{2}+q_{3}^{2}\right) & 2\left(q_{0}q_{1}+q_{2}q_{3}\right)\\ 2\left(q_{0}q_{2}+q_{1}q_{3}\right) & 2\left(q_{2}q_{3}-q_{0}q_{1}\right) & 1-2\left(q_{1}^{2}+q_{2}^{2}\right) \end{array}\right)$$

(A19) $$H_{12}=-g\;\left(\;\begin{array}{cccc} -q_{2} & q_{3} & -q_{0} & q_{1}\\ q_{1} & q_{0} & q_{3} & q_{2}\\ q_{0} & -q_{1} & -q_{2} & q_{3} \end{array}\right)$$

(A20) $$H_{22}=h\;\left\{\sin\alpha \left(\begin{array}{cccc} q_{0} & q_{1} & -q_{2} & -q_{3}\\ -q_{3} & q_{2} & q_{1} & -q_{0}\\ q_{2} & q_{3} & q_{0} & q_{1} \end{array}\right)\right.+\left.\cos\alpha \left(\begin{array}{cccc} -q_{2} & q_{3} & -q_{0} & q_{1}\\ q_{1} & q_{0} & q_{3} & q_{2}\\ q_{0} & -q_{1} & -q_{2} & q_{3} \end{array}\right)\right\}$$

where $q_{0}$, $q_{1}$, $q_{2}$ and $q_{3}$ are the components of quaternion ${}^{e}q$, *g* represents gravity, α is the angle between the local magnetic and gravity fields and *h* is the magnetic field modulus [14].
